# Survival Outcomes and Tumor IMP3 Expression in Patients with Sarcomatoid Metastatic Renal Cell Carcinoma

**DOI:** 10.1155/2015/181926

**Published:** 2015-01-20

**Authors:** Srinivas K. Tantravahi, Daniel Albertson, Archana M. Agarwal, Sowmya Ravulapati, Austin Poole, Shiven B. Patel, Jamil S. Hawatmeh, Alli M. Straubhar, Ting Liu, David D. Stenehjem, Neeraj Agarwal

**Affiliations:** ^1^Department of Internal Medicine, University of Utah Huntsman Cancer Institute, Salt Lake City, UT 84112, USA; ^2^ARUP Laboratories, Department of Pathology, The University of Utah, Salt Lake City, UT 84108, USA; ^3^Pharmacotherapy Outcomes Research Center (PORC), College of Pharmacy, The University of Utah, Salt Lake City, UT 84112, USA

## Abstract

Metastatic renal cell carcinoma with sarcomatoid histology (SmRCC) is associated with poor survival. No data is available from randomized trials on the efficacy of vascular endothelial growth factor (VEGF) and mammalian target of rapamycin (mTOR) inhibitors in SmRCC. We identified SmRCC patients from a single institutional database. To identify predictive and prognostic biomarkers, immunohistochemistry (IHC) analysis was performed on the tumor samples for downstream targets of VEGF and mTOR pathways. Survival outcomes were stratified by IHC analysis, extent of sarcomatoid component, Memorial Sloan-Kettering Cancer Center (MSKCC), and Heng risk criteria. Twenty-seven patients with SmRCC were included. First line therapy included targeted therapy (*n* = 19), immunotherapy (*n* = 4), cytotoxic chemotherapy (*n* = 1), and no treatment (*n* = 3). Median OS was 8.2 months (95% CI 3.8–14.2 months). Median survival in months, based on MSKCC and Heng risk groups, was favorable 89.3 versus 84.5, intermediate 9.5 versus 12.7, and poor 3.9 versus 5.1. None of the IHC markers predicted outcomes of treatment with VEGF or mTOR inhibitors. Only tumor IMP3 expression was associated with inferior OS, although not statistically significant (IMP3 negative 14.2 versus IMP3 positive 4.9 months; HR 0.46, 95% CI 0.16–1.21; *P* = 0.12). The study was limited by small sample size.

## 1. Introduction

It is estimated that, in 2014, renal cell carcinoma (RCC) accounted for approximately 64,000 new cancer cases and 14,000 deaths in the US [1]. Clear cell histology accounts for over 75% of RCCs and other variants account for the rest of the cases [2].

The advances in understanding of the pathogenesis of clear cell variant metastatic RCC (cc mRCC) have revolutionized its treatment with development of drugs targeting angiogenesis and mammalian target of rapamycin (mTOR) pathways. Over the past decade, seven new drugs were approved for clinical use in cc mRCC including tyrosine kinase inhibitors (TKIs) of the vascular endothelial growth factor (VEGF) receptor (sorafenib [3], sunitinib [4], pazopanib [5], and axitinib [6]), VEGF ligand inhibitor (bevacizumab [7]), and mTOR inhibitors (temsirolimus [8] and everolimus [9]). While the treatment landscape of cc mRCC has advanced dramatically, there has not been a single drug approved specifically for other variants of mRCC.

Sarcomatoid variant can coexist with other variants in approximately 5% to 8% of mRCCs and its presence is associated with an aggressive clinical course and a poor prognosis [10–12]. The identification of both epithelial and mesenchymal elements defines the histologic diagnosis of sarcomatoid differentiation. Studies based on chromosomal alterations and X-chromosome inactivation confirmed the common cell of origin for both sarcomatoid and epithelial components, but the clonal evolution and divergence leads to the epithelial mesenchymal transition [13, 14]. Treatment of SmRCC is poorly defined and is largely based on retrospective data from small case series and single institutional experiences. Cytotoxic chemotherapy was considered a rational approach due to the analogy to conventional sarcomas. However, the results from two prospective clinical trials evaluating cytotoxic chemotherapy with doxorubicin and gemcitabine [15] and doxorubicin and ifosfamide [16] were disappointing with median survival of 3.9 months and 8.8 months, respectively. In another prospective study, sorafenib has shown antitumor activity in 5 of 9 patients who progressed on doxorubicin and gemcitabine combination chemotherapy [17]. Several single institutional retrospective reports indicated the potential value of targeted therapies with VEGF TKIs and mTOR inhibitors in SmRCC. One study reported a median OS of 11.8 months with VEGF TKIs [18] and, in a more recent study, a median OS of 15.7 months was reported with sunitinib [19]. The therapeutic utility of mTOR inhibition in this subset of patients is not clear; one study reported a median OS of 8.2 months in SmRCC patients treated with temsirolimus or everolimus [20].

Another area of exploration in SmRCC is the identification of predictive and prognostic biomarkers to treatment response and overall survival. Presence of certain histologic features such as necrosis and microvascular invasion is associated with poor prognosis [10, 11]. Additionally, expression of c-KIT by immunohistochemistry (IHC) was found to be associated with improved response rates to sorafenib and longer survival, although the study was limited by a small sample size [21]. In an effort to identify predictive biomarkers for the currently approved therapeutic agents for mRCC, we conducted IHC analysis of tumor samples for downstream targets of vascular endothelial growth factor (VEGF) and mammalian target of rapamycin pathways and correlated IHC analysis with survival outcomes. Here, we report the treatment and survival outcomes of patients with SmRCC in correlation with degree of sarcomatoid component, MSKCC risk group, Heng risk group, and aforementioned IHC markers.

## 2. Methods

### 2.1. Patients

From a single institutional database (years 2000–2012), all patients with SmRCC were included. Clinical characteristics and treatments received were assessed by chart review. Vital status was obtained from the social security death index. The University of Utah Institutional Review Board (IRB number 67518) approved the study protocol.

### 2.2. Pathology and Immunohistochemistry Analysis

Except in one patient, primary nephrectomy samples were assessed for diagnosis of SmRCC. In one patient who had not undergone prior nephrectomy, diagnosis was confirmed by core biopsies of the metastatic disease site. Two independent pathologists (Daniel Albertson and Ting Liu) estimated the degree of sarcomatoid component and reported the IHC analysis as outlined below. Sarcomatoid predominant and nonpredominant variants were defined by the sarcomatoid component of ≥20% or <20%, respectively [18, 22]. IHC staining was performed on 4-micron thick sections of formalin-fixed, paraffin-embedded tissues. Sections were air-dried, deparaffinized, and stained at 37°C on the automated immunostainer (BenchMark Ultra) from Ventana Medical Systems, Tucson, AZ. Following staining, tissue was dehydrated, cleared in xylene, and cover-slipped. Positive and negative controls were run with each batch of slides. All slides were prepared at the University of Utah/ARUP Laboratories, Salt Lake City, UT. IHC analysis of the tumor samples was performed for proviral integration site proteins (PIM 1,2,3), phosphorylated mammalian target of rapamycin (Phos-mTOR) signaling, phosphorylated ribosomal protein S6 (phos6Rib), integrase interactor 1 (INI-1), insulin-like growth factor II mRNA-binding protein 3 (IMP3), phosphatase and tensin homolog (PTEN), beta-catenin (Bcat), E-cadherin (Ecad), p53, and epithelial membrane antigen (EMA), where available. INI-1 staining was scored as retained (1) or lost (0). All additional staining results were scored 0–4 (0–5% = 0, 6–20% = 1, 21–50% = 2, 51–75% = 3, and 76–100% = 4); 0-1 score was considered as negative and 2–4 as positive.

### 2.3. Statistical Analysis

Median overall survival was assessed by Kaplan-Meier method with stratified log rank by IHC analysis, extent of sarcomatoid component, MSKCC risk criteria, and Heng risk criteria. Cox proportional hazards models were used to determine hazard ratios (HR) and 95% confidence intervals (CI).

## 3. Results

### 3.1. Patient Characteristics

The cohort included 27 patients, 18 men (67%) and nine women (33%), with a median age of 63 years (range 39–74 years) (Table 1). Twenty-four patients received systemic therapy, two patients died shortly after nephrectomy due to progressive disease, and one patient was lost to follow-up. First line treatment (*n* = 24) included targeted therapy (*n* = 19), immunotherapy (*n* = 4), and cytotoxic chemotherapy (*n* = 1). Two additional patients who received first line immunotherapy subsequently received targeted therapy at progression. Patients belonged to the following MSKCC risk groups: 2 (7%) favorable, 18 (67%) intermediate, and 7 (26%) poor risk. Heng risk group stratification was as follows: 1 (4%) favorable, 17 (63%) intermediate, and 9 (33%) poor risk. IHC analysis was performed in 21 tumor samples and in 6 patients tissue was not available. Comprehensive IHC analysis was performed in 17 patients and IMP3 was analyzed in 4 additional patients due to limited tissue availability.

### 3.2. Clinical Outcome

**Figure 1:**
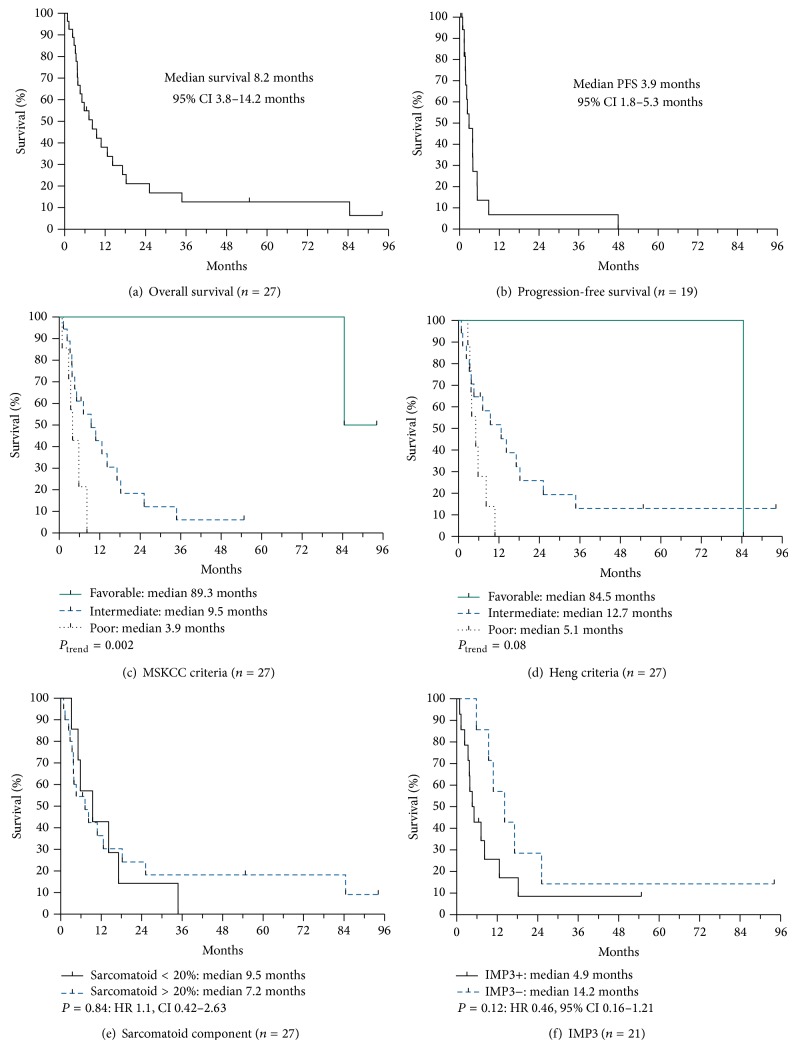
Survival outcomes in patients with sarcomatoid metastatic renal cell carcinoma (SmRCC). (a) Overall survival in SmRCC. (b) Progression-free survival of treated SmRCC. Overall survival stratified by MSKCC (c) and Heng risk criteria (d), extent of sarcomatoid component (e), and IMP3 staining (f).

Median overall survival of the cohort was 8.2 months (95% CI 3.8–14.2 months, Figure 1(a)). Median OS in those who received immunotherapy was 12.5 months, VEGFR-TKI 8.2 months, mTOR inhibitors 3.7 months, and no treatment 3.7 months. Median PFS was 2.9 months (95% CI 1.8–5.3 months, Figure 1(b)). An objective response was achieved in 12% of treated patients (*n* = 3).

### 3.3. Survival by Prognostic Criterion

Median OS based on MSKCC risk criteria (Figure 1(c)) was favorable 89.3 months, intermediate 9.5 months, and poor 3.9 months; *P*_trend_ = 0.002. Similarly, median OS based on Heng risk criteria (Figure 1(d)) was as follows; favorable 84.5 months, intermediate 12.7 months, and poor 5.1 months; *P*_trend_ = 0.08. Degree of sarcomatoid component did not influence survival (Figure 1(e)). Median OS in sarcomatoid nonpredominant patients was 9.5 months compared to 7.2 months in predominant patients (HR 1.1, 95% CI 0.42–2.63; *P* = 0.84).

### 3.4. Outcomes of Pathology and Immunohistochemistry Analysis

Of all the markers evaluated by IHC, only IMP3 expression was associated with overall survival (Supplementary Table 1 in Supplementary Material available online at http://dx.doi.org/10.1155/2015/181926). Of 21 patients in whom IMP3 analysis was available, the median survival in IMP3 positive patients (*n* = 14, 67%) was 4.9 months versus 14.2 months in negative patients (7, 33%); however, the difference was not statistically significant (IMP3− versus IMP3+: HR 0.46, 95% CI 0.16–1.21; *P* = 0.12, Figure 1(f)). The median PFS of TKI or mTOR inhibitor treated patients was 4.0 months in IMP3 positive patients versus 3.9 months in IMP3 negative patients; *P* = 0.49. In IMP3 positive patients no difference in PFS was observed in patients treated with an mTOR inhibitor (*n* = 3, 3.8 months) versus a TKI (*n* = 9, 4 months; *P* = 0.71). The median sarcomatoid percentage in IMP3 negative and positive patients was 10% versus 50%, respectively; *P* = 0.14. In IMP3 positive patients (*n* = 14) all patients except one were considered sarcomatoid predominant (*n* = 13, 93%), whereas sarcomatoid nonpredominant features were observed in 4 of 7 (57%) IMP3 negative patients. This correlation with sarcomatoid predominant and IMP3 positivity was statistically significant; *P* = 0.03.

## 4. Discussion

Despite significant advances in the field of mRCC with the advent of targeted therapy, the clinical outcomes in patients with SmRCC remain poor. More importantly, the prognostic utility of MSKCC and Heng risk stratification in this subset of patients is less clear. Recently Pal et al. reported inferior survival in poor risk patients by MSKCC and Heng criteria in comparison to intermediate risk patients [22]. Our study also supports this observation indicating the value of current prognostic models of mRCC in this subset of patients. Another area of controversy in this subset of patients is the prognostic value of the percentage or degree of sarcomatoid component. Prior studies in the pretargeted therapy era have shown aggressive clinical course and poor survival with increasing degree of sarcomatoid component [10, 11]. One study reported significantly improved median survival (11.8 months) in patients treated with VEGF TKIs with sarcomatoid component of <20% with objective responses in half of the patients in comparison to a median OS of 5.3 months in patients with >20% [18]. However, subsequent studies failed to show similar differences in survival based on the percentage of sarcomatoid component [19, 22]. In our current analysis, the percentage of sarcomatoid component using cutoff of 20% was not predictive of response to treatment or survival.

Currently there is no uniform consensus on optimal treatment for sarcomatoid mRCC. Two prospective clinical trials evaluating cytotoxic chemotherapy yielded disappointing results. One study including 27 patients with SmRCC treated with doxorubicin and ifosfamide reported a median survival of 3.9 months with no objective responses [15]. Similarly, in a second study conducted by the Eastern Cooperative Oncology Group, the combination of doxorubicin and gemcitabine in 39 patients with advanced SmRCC resulted in an objective response in 6 patients (16%) and stable disease in ten (26%) patients. The median OS and PFS were 8.8 and 3.5 months, respectively [16]. Although prospective trials of systemic chemotherapy in SmRCC yielded disappointing results, in a systematic literature review of all published clinical trials of chemotherapy in mRCC between January 2003 and November 2014, combination of doxorubicin and gemcitabine or capecitabine has shown promising antitumor activity in mRCC with sarcomatoid differentiation [23]. A prospective open label study evaluated the efficacy of sorafenib following disease progression on prior chemotherapy. Fifteen patients with SmRCC were treated with gemcitabine and doxorubicin for a median of four monthly cycles of chemotherapy (range 1–7 cycles) and then switched to treatment with sorafenib upon disease progression. Median time to progression on chemotherapy was 6.6 months (range 0.8 to 8 months). Six patients died due to progressive disease prior to being switched to sorafenib. The median time to progression on sorafenib was 10.9 months (range 0.6 to 25.5 months), suggesting that a subset of patients may benefit from a sequential therapy with chemotherapy followed by a VEGF TKI [17]. In addition, several retrospective studies also support the value of VEGF TKIs and mTOR inhibitors in SmRCC [18–20]. One study reported a median survival of 11.8 months with VEGF TKI based therapy [18], while another study reported a median OS of 15.7 months with sunitinib [19]. In a recently reported single institutional retrospective study, by Pal and colleagues, of 21 patients with SmRCC treated with different agents (VEGF TKIs, immunotherapy, and chemotherapy) the median OS was 18 months [22]. In our analysis, the median OS was lower (8.2 months) compared to Pal et al. (2013). This difference in survival is likely due to sample and selection bias.

Given the lack of approved agents, therapies inhibiting VEGF and mTOR pathways are used in the treatment of SmRCC, based on extrapolation of data from randomized trials in cc mRCC. There is a need to identify prognostic and predictive biomarkers to these therapies (VEGF inhibitors versus mTOR inhibitors) in SmRCC. Thus, we conducted IHC analysis of tumor samples for downstream targets VEGF and mammalian target of rapamycin pathways and correlated with treatment and survival outcomes in our patients with SmRCC. In our current analysis, none of the IHC markers predicted response to treatment with either VEGF or mTOR inhibitors. Only IMP3 expression had a trend for association with poor overall survival. IMP3 is an oncofetal protein belonging to the family of insulin-like growth factor II (IGF-II) mRNA-binding protein (IMP) that also includes IMP1 and IMP2 [24]. The biologic function of IMP family of proteins in early embryogenesis is well established. Intriguingly, IMP3 expression is undetectable in most adult tissues, but high-level expression has been found in malignant tumors [24]. The role of IMP3 in tumor cell proliferation and invasion is further supported by the fact that its expression is associated with an aggressive phenotype with increased risk of progression to metastatic disease in localized renal cell cancers with clear cell and non-clear cell histology [25, 26]. Notably, another independent follow-up study including a large number of patients with clear cell RCC confirmed IMP3 as an independent prognostic factor associated with advanced disease and increased incidence of coagulative necrosis and sarcomatoid differentiation. In addition, patients with positive IMP3 tumors on IHC were 5 times more likely to die in comparison to IMP3 negative tumors (HR 4.60, 95% CI 3.53–6.00; *P* < 0.001) [27]. However to our knowledge, no studies have been reported on the prognostic value of IMP3 expression specifically in SmRCC. In our current study of patients with SmRCC, patients with IMP3 positive tumors had inferior median OS; however this did not reach statistical significance presumably due to small sample size. IMP3 positive tumors did have a significantly higher percentage of sarcomatoid differentiation. No difference in progression-free survival with targeted therapy (VEGFR-TKIs and mTOR inhibitors) was observed based on IMP3 expression. These results suggest the potential prognostic value of tumor IMP3 expression in SmRCC patients. Further validation in a larger sample is necessary to confirm our findings.

## 5. Conclusion

SmRCC is associated with inferior outcomes with currently approved treatments for mRCC. MSKCC and Heng risk stratification hold their prognostic value in these patients with SmRCC. Percentage of sarcomatoid component directly correlated with IMP3 expression. Tumor IMP3 expression is associated with inferior survival. In our cohort the percentage of sarcomatoid component using the traditional cutoff of 20% did not correlate with survival. The main limitation of this study is the small sample size due to the rarity of disease. Lack of uniform reporting of sarcomatoid differentiation is a major concern for identification of this subset of patients. We overcame this issue by reassessment of pathology samples by two independent genitourinary pathologists to confirm the presence and estimate the degree of sarcomatoid differentiation. Another limitation is that laboratory and imaging studies were not performed at predetermined intervals, due to the retrospective nature of study. However all patients received standard of care treatment as per our institutional guidelines. Further validation of these findings is needed in a larger cohort of patients with SmRCC.

## Supplementary Material

The immunohistochemistry staining of the tumor tissue was performed for downstream targets of vascular endothelial growth factor (VEGF) and mammalian target of rapamycin (mTOR) pathways and the scores of all patients (1 to 27) are outlined in the Supplementary Table 1.

## Figures and Tables

**Table 1:** Demographics of the sarcomatoid metastatic renal cell carcinoma cohort.

	Number (%)
Median age (range)	63 (39–74 years)
Sex	
Male	18 (67%)
Female	9 (33%)
Histologic subtype	
Sarcomatoid	4 (15%)
Sarcomatoid + clear^†^	19 (70%)
Sarcomatoid + other^‡^	4 (15%)
Metastatic sites	22 (81%)
Lung	11 (42%)
Bone	6 (22%)
Brain	5 (18%)
Liver	26 (96%)
Prior nephrectomy	
MSKCC risk group	
Favorable	2 (7%)
Intermediate	18 (67%)
Poor	7 (26%)
Heng risk group	
Favorable	1 (4%)
Intermediate	17 (63%)
Poor	9 (33%)
Sarcomatoid features	
Predominant	20 (74%)
Nonpredominant	7 (26%)
Median sarcomatoid % (IQR)	50 (15–80%)
IMP3	
Positive	14 (67%)
Negative	7 (33%)

^†^One patient demonstrated both clear cell and papillary histology.

^‡^Papillary (*n* = 1), granular cell (*n* = 1), and unclassified types (*n* = 2).

MSKCC, Memorial Sloan-Kettering Cancer Center; IMP-3, insulin-like growth factor II mRNA-binding protein 3; IQR, interquartile range.

## References

[1] Siegel R., Ma J., Zou Z., Jemal A. (2014). Cancer statistics, 2014. *CA Cancer Journal for Clinicians*.

[2] Lopez-Beltran A., Carrasco J. C., Cheng L., Scarpelli M., Kirkali Z., Montironi R. (2009). 2009 update on the classification of renal epithelial tumors in adults. *International Journal of Urology*.

[3] Escudier B., Eisen T., Stadler W. M. (2007). Sorafenib in advanced clear-cell renal-cell carcinoma. *The New England Journal of Medicine*.

[4] Motzer R. J., Hutson T. E., Tomczak P. (2007). Sunitinib versus interferon alfa in metastatic renal-cell carcinoma. *The New England Journal of Medicine*.

[5] Sternberg C. N., Davis I. D., Mardiak J. (2010). Pazopanib in locally advanced or metastatic renal cell carcinoma: results of a randomized phase III trial. *Journal of Clinical Oncology*.

[6] Rini B. I., Escudier B., Tomczak P. (2011). Comparative effectiveness of axitinib versus sorafenib in advanced renal cell carcinoma (AXIS): a randomised phase 3 trial. *The Lancet*.

[7] Yang J. C., Haworth L., Sherry R. M. (2003). A randomized trial of bevacizumab, an anti-vascular endothelial growth factor antibody, for metastatic renal cancer. *The New England Journal of Medicine*.

[8] Hudes G., Carducci M., Tomczak P. (2007). Temsirolimus, interferon alfa, or both for advanced renal-cell carcinoma. *The New England Journal of Medicine*.

[9] Motzer R. J., Escudier B., Oudard S. (2008). Efficacy of everolimus in advanced renal cell carcinoma: a double-blind, randomised, placebo-controlled phase III trial. *The Lancet*.

[10] de Peralta-Venturina M., Moch H., Amin M. (2001). Sarcomatoid differentiation in renal cell carcinoma: a study of 101 cases. *The American Journal of Surgical Pathology*.

[11] Cheville J. C., Lohse C. M., Zincke H. (2004). Sarcomatoid renal cell carcinoma: an examination of underlying histologic subtype and an analysis of associations with patient outcome. *American Journal of Surgical Pathology*.

[12] Mian B. M., Bhadkamkar N., Slaton J. W. (2002). Prognostic factors and survival of patients with sarcomatoid renal cell carcinoma. *Journal of Urology*.

[13] Dal Cin P., Sciot R., Van Poppel H., Balzarini P., Roskams T., Van den Berghe H. (2002). Chromosome changes in sarcomatoid renal carcinomas are different from those in renal cell carcinomas. *Cancer Genetics and Cytogenetics*.

[14] Jones T. D., Eble J. N., Wang M., Maclennan G. T., Jain S., Cheng L. (2005). Clonal divergence and genetic heterogeneity in clear cell renal cell carcinomas with sarcomatoid transformation. *Cancer*.

[15] Escudier B., Droz J. P., Rolland F. (2002). Doxorubicin and ifosfamide in patients with metastatic sarcomatoid renal cell carcinoma: a phase II study of the genitourinary group of the French federation of cancer centers. *The Journal of Urology*.

[16] Haas N. B., Lin X., Manola J. (2012). A phase II trial of doxorubicin and gemcitabine in renal cell carcinoma with sarcomatoid features: ECOG 8802. *Medical Oncology*.

[17] Staehler M., Haseke N., Roosen A. (2010). Sorafenib after combination therapy with gemcitabine plus doxorubicine in patients with sarcomatoid renal cell carcinoma: a prospective evaluation. *European Journal of Medical Research*.

[18] Golshayan A. R., George S., Heng D. Y. (2009). Metastatic sarcomatoid renal cell carcinoma treated with vascular endothelial growth factor-targeted therapy. *Journal of Clinical Oncology*.

[19] Kunene V., Miscoria M., Pirrie S., Islam M. R., Afshar M., Porfiri E. (2014). Sarcomatoid renal cell carcinoma: clinical outcome and survival after treatment with sunitinib. *Clinical Genitourinary Cancer*.

[20] Voss M. H., Bastos D. A., Karlo C. A. (2014). Treatment outcome with mTOR inhibitors for metastatic renal cell carcinoma with nonclear and sarcomatoid histologies. *Annals of Oncology*.

[21] Zhang H. L., Zhu Y., Qin X. J. (2013). c-KIT: potential predictive factor for the efficacy of sorafenib in metastatic renal cell carcinoma with sarcomatoid feature. *Clinical Genitourinary Cancer*.

[22] Pal S. K., Jones J. O., Carmichael C. (2013). Clinical outcome in patients receiving systemic therapy for metastatic sarcomatoid renal cell carcinoma: a retrospective analysis. *Urologic Oncology*.

[23] Buti S., Bersanelli M., Sikokis A. (2013). Chemotherapy in metastatic renal cell carcinoma today? A systematic review. *Anti-Cancer Drugs*.

[24] Nielsen J., Christiansen J., Lykke-Andersen J., Johnsen A. H., Wewer U. M., Nielsen F. C. (1999). A family of insulin-like growth factor II mRNA-binding proteins represses translation in late development. *Molecular and Cellular Biology*.

[25] Jiang Z., Chu P. G., Woda B. A. (2006). Analysis of RNA-binding protein IMP3 to predict metastasis and prognosis of renal-cell carcinoma: a retrospective study. *The Lancet Oncology*.

[26] Jiang Z., Lohse C. M., Chu P. G. (2008). Oncofetal protein IMP3: a novel molecular marker that predicts metastasis of papillary and chromophobe renal cell carcinomas. *Cancer*.

[27] Hoffmann N. E., Sheinin Y., Lohse C. M. (2008). External validation of IMP3 expression as an independent prognostic marker for metastatic progression and death for patients with clear cell renal cell carcinoma. *Cancer*.

