# Supplementary material for: Survival Outcomes and Tumor IMP3 Expression in Patients with Sarcomatoid Metastatic Renal Cell Carcinoma
Source: J Oncol. 2015 Jan 20;2015:181926. doi: 10.1155/2015/181926 (PMC4320862; doi:10.1155/2015/181926)
Supplement: Supplementary file 1 — The immunohistochemistry staining of the tumor tissue was performed for downstream targets of vascular endothelial growth factor (VEGF) and mammalian target of rapamycin (mTOR) pathways and the scores of all patients (1 to 27) are outlined in the Supplementary Table 1. [file 181926.f1.pdf]

Supplementary Table 1: Immunohistochemistry staining results

S. No	PIM2	PIM2	PIM3	Phos-MTOR	Phos-S6Rib	INI-1	IMP3	PTEN	Bcat	Ecad	p53	EMA
1	0	3	0	2	3	1	4	4	4	0	3	3
2	1	3	0	2	2	1	3	0	4	0	1	2
3	0	2	0	4	4	1	3	3	3	3	0	2
4	1	2	2	2	0	1	0	2	4	0	0	0
5	4	4	2	1	0	1	2	2	4	3	1	3
6	0	0	0	2	4	1	3	0	2	1	0	3
7	0	2	4	3	3	1	0	3	4	4	0	1
8	0	0	0	3	4	1	0	0	4	0	0	3
9	1	3	0	4	4	1	4	3	4	0	0	0
10	0	0	1	3	2	1	2	0	0	0	1	2
11	0	0	0	3	4	1	0	0	4	0	0	0
12	0	2	0	4	1	1	4	0	4	0	0	0
13	0	4	1	3	4	1	3	4	4	0	3	0
14	0	2	0	1	4	1	3	0	4	0	0	0
15	0	0	0	3	2	1	0	0	4	0	3	0
16	3	3	1	4	3	1	0	3	4	2	0	0
17	0	3	3	4	3	1	4	0	4	0	0	4
18	N/A	N/A	N/A	N/A	N/A	N/A	0	N/A	N/A	N/A	N/A	N/A
19*	N/A	N/A	N/A	N/A	N/A	N/A	2	N/A	N/A	N/A	N/A	N/A
20*	N/A	N/A	N/A	N/A	N/A	N/A	2	N/A	N/A	N/A	N/A	N/A
21*	N/A	N/A	N/A	N/A	N/A	N/A	4	N/A	N/A	N/A	N/A	N/A
22	N/A	N/A	N/A	N/A	N/A	N/A	N/A	N/A	N/A	N/A	N/A	N/A
23	N/A	N/A	N/A	N/A	N/A	N/A	N/A	N/A	N/A	N/A	N/A	N/A
24	N/A	N/A	N/A	N/A	N/A	N/A	N/A	N/A	N/A	N/A	N/A	N/A
25	N/A	N/A	N/A	N/A	N/A	N/A	N/A	N/A	N/A	N/A	N/A	N/A
26	N/A	N/A	N/A	N/A	N/A	N/A	N/A	N/A	N/A	N/A	N/A	N/A
27	N/A	N/A	N/A	N/A	N/A	N/A	N/A	N/A	N/A	N/A	N/A	N/A

[†]Immunohistochemistry staining was performed only for IMP3 due to limited tissue availability.

N/A: Tissue not available for immunohistochemistry analysis

Abbreviations: PIM, proviral integration site proteins; Phos-mTOR, phosphorylated mammalian target of rapamycin signaling; phos6Rib, phosphorylated ribosomal protein S6; INI-1, integrase interactor 1; IMP-3, Insulin-like growth factor II mRNA-binding protein 3; PTEN, phosphatase and tensin homolog; Bcat, Beta-catenin; Ecad, E-cadherin; EMA, epithelial membrane antigen. Stain score 0-4 (0-5%=0, 6-20%=1, 21-50%=2, 51-75%=3, 76-100%=4); 0-1 was considered as negative and 2-4 as positive.
